# IL-1R2 promotes tumorigenesis and modulates the tumor immune microenvironment in colorectal cancer

**DOI:** 10.1007/s00262-025-04138-5

**Published:** 2025-08-06

**Authors:** Yanyan Lang, Hao Huang, Hongwei Jiang, Shaoxian Wu, Zhang Fang, Dachuan Zhang, Heya Qian, Yingting Liu, Maoling Yuan, Bin Xu, Lujun Chen, Xiao Zheng, Jingting Jiang

**Affiliations:** 1 Department of Tumor Biological Treatment, The Third Affiliated Hospital of Soochow University, Jiangsu, 213003 Changzhou China; 2 Jiangsu Engineering Research Center for Tumor Immunotherapy, The Third Affiliated Hospital of Soochow University, Jiangsu, 213003 Changzhou China; 3 Institute of Cell Therapy, The Third Affiliated Hospital of Soochow University, Jiangsu, 213003 Changzhou China

**Keywords:** Interleukin-1 receptor 2, Colorectal cancer, Immune checkpoint, CTLA-4, PD-1

## Abstract

Colorectal cancer (CRC) continues to be a major global health challenge due to its high incidence and mortality rates, emphasizing the critical need for innovative therapeutic strategies. IL-1R2, a member of the IL-1 receptor family, plays a pivotal role in both tumorigenesis and antitumor immunity. However, its precise role in tumor development and its impact on immune checkpoint inhibitors (ICIs) therapy in CRC remain poorly understood. We examined tumor progression in wild-type and IL-1R2-deficient mice using an AOM/DSS-induced colitis-associated colorectal cancer model treated with combined ICIs therapy. Our findings revealed that IL-1R2 deficient mice exhibited a significant reduction in tumor burden, accompanied by alterations in the carcinogenic program and enhanced immunogenicity of tumor cells. Furthermore, the deletion of IL-1R2 resulted in an increased proportion of exhausted CD8^+^ T cells, a population commonly enriched for tumor antigen-specific T cells, suggesting an augmentation of tumor-specific immune responses. Moreover, IL-1R2 deletion upregulated genes linked to antigen presentation in dendritic cells, indicating enhanced activation of the adaptive immune system. Collectively, these findings position IL-1R2 as a promising therapeutic target for improving the efficacy of treatment strategies in CRC.

**Supplementary Information:**

The online version contains supplementary material available at 10.1007/s00262-025-04138-5.

## Introduction

Colorectal cancer (CRC) ranks as the third most common malignancy and is the second leading cause of cancer-related deaths globally^1^. Despite advancements in early screening and diagnostic tools that have improved the detection of early-stage tumors, a significant proportion of patients are still diagnosed at advanced stages. This late diagnosis greatly compromises treatment efficacy and negatively impacts patient survival outcomes^2^. Current treatment options for CRC encompass surgery, radiotherapy, chemotherapy, targeted therapy, and immunotherapy^3^. Among these, immunotherapy has emerged as a groundbreaking approach by harnessing the body’s immune system to enhance its tumor-killing capabilities. Immune checkpoint inhibitors (ICIs) have demonstrated transformative efficacy in treating microsatellite instability-high (MSI-H) CRC, significantly improving outcomes for this subset of patients^4^. However, the therapeutic effectiveness of ICIs in microsatellite-stable (MSS) CRC—which accounts for the majority of CRC cases—has been limited when used as monotherapy^5^. Recent clinical advancements are shedding light on strategies to enhance the efficacy of ICIs in MSS CRC. For instance, in a phase Ib clinical trial, the combination of a PD-1 inhibitor and the anti-angiogenic agent regorafenib achieved an objective response rate (ORR) of 33% in MSS CRC patients, demonstrating the potential of synergistic therapeutic approaches^6^. Additionally, IBI363, a PD-1/IL-2^α−bias^ bispecific antibody, has shown promise by selectively activating tumor-specific PD-1^+^CD25^+^ T cells while minimizing effects on bystander T cells. In clinical evaluations, IBI363 demonstrated manageable safety and achieved an ORR of 12.7% in the overall population, including 13.2% in patients with liver metastases, highlighting its potential benefit in these challenging cases 7. These findings highlight the importance of combination therapies and innovative drug designs targeting multiple pathways.

Chronic inflammation is a critical driver of CRC development, with interleukin-1 (IL-1) serving as a key pro-inflammatory cytokine that regulates intestinal inflammation and tumor progression^8^. To maintain immune homeostasis and prevent excessive inflammation, the body has evolved sophisticated regulatory mechanisms to control IL-1 signaling. One such mechanism involves IL-1R2, a member of the IL-1 receptor family, which functions as a "decoy receptor"^9^. IL-1R2 binds to IL-1 without triggering downstream signaling, thereby dampening the pro-inflammatory effects of IL-1 and modulating the inflammatory response in the intestinal microenvironment. This receptor is expressed across various immune cell types, including neutrophils, dendritic cells, monocytes, macrophages, and regulatory T (Treg) cells^10^–^13^. We have demonstrated that the deletion of IL-1R2 specifically in Treg cells leads to enhanced antitumor immunity^14^. Beyond its role as a decoy receptor, IL-1R2 also functions intracellularly to modulate inflammation. For instance, it has been shown to interact with the transcription factor c-Fos in colon tumor cells, promoting the expression of vascular endothelial growth factor (VEGF) and IL-6, which in turn enhances the proliferation and migration of colon cancer cells^15^. Additionally, the intracellular domain of IL-1R2 can associate with ubiquitin-specific protease 15 (USP15), forming a complex that stabilizes the protein levels of B-cell-specific Moloney murine leukemia virus integration site 1 (BMI1). This stabilization supports the self-renewal capacity of cancer stem cells^16^. Collectively, these findings underscore the multifaceted role of IL-1R2 in regulating tumor progression and antitumor immune responses, highlighting its potential as a therapeutic target in CRC.

However, the role of IL-1R2 in immune checkpoint blockade therapy for colon cancer remains largely unexplored. In this study, we investigated the role and underlying mechanisms of IL-1R2 in regulating immune checkpoint blockade therapy in vivo. Specifically, we examined tumor progression in wild-type and IL-1R2 knockout mice in a colitis-associated colorectal cancer model induced by AOM/DSS. Additionally, we treated these mice with ICIs to assess tumor growth under these therapeutic conditions. To gain deeper mechanistic insights, we employed single-cell RNA sequencing and a series of immunological assays, aiming to identify novel strategies to improve immunotherapy for colon cancer.

## Result

### Lack of IL-1R2 plus ICI therapy inhibits tumor growth

**Fig. 1:**
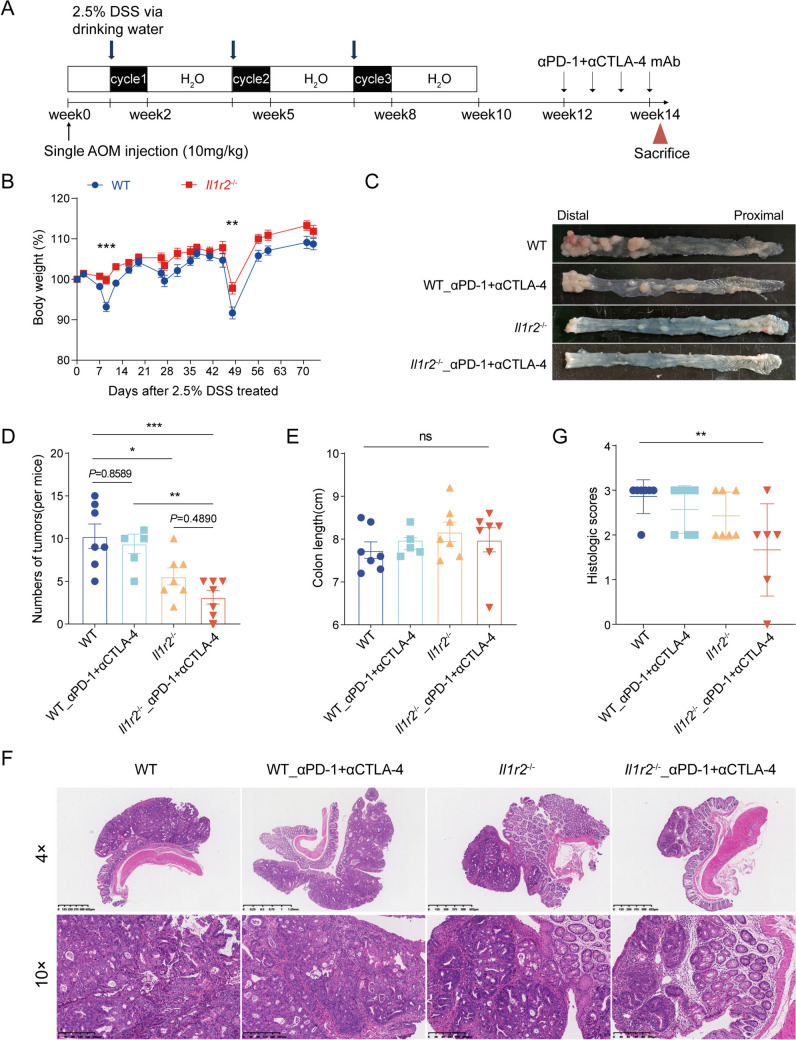
Enhanced efficacy of immune checkpoint blockade with IL-1R2 deletion. **a** AOM/DSS treatment flow chart. Anti-PD-1 antibody and anti-CTLA-4 antibody were administered as indicated by the arrow, every 4 days for four treatments. **b** Body weight curves of female mice in different group during AOM/DSS treatment. n = 14 per group. **c** Macroscopic representative images of mouse tumors at sacrifice. **d** Statistical plot of tumor number in female mice at sacrifice. n = 5–7 per group. **e** Statistical plot of colon length in female mice at sacrifice. n = 5–7 per group. **f** Representative hematoxylin and eosin (H&E) staining of tumor tissue from control mice and Il1r2^−/−^ mice at the end of combined treatment. (4 × and 10 ×) n = 5–7 per group. **g** Histopathological scores were performed on the tumors of the four groups of mice according to the degree of hyperplasia. n = 5–7 per group. Data are presented as Mean ± SEM, *P < 0.05, **P < 0.01, ***P < 0.001, ****P < 0.0001

To explore the role of IL-1R2 in immune checkpoint blockade therapy, IL-1R2 knockout (Il1r2^−/−^, KO) and wild-type (WT) female mice were treated with AOM/DSS to induce tumor formation. Two weeks after the end of induction, we started treatment with a combination of anti-PD-1 and anti-CTLA-4 monoclonal antibodies, administered every four days for four doses (Fig. 1a). After two weeks, the mice were assessed for weight loss and tumor growth. IL-1R2 knockout mice exhibited less weight loss compared to WT mice (Fig. 1b), suggesting reduced inflammation in the knockout group. We also measured colon length, a common indicator of inflammatory status; however, no significant differences were observed between the two groups of mice, regardless of treatment (Fig. 1e). Under untreated conditions, Il1r2^−/−^ mice had fewer tumors than WT mice. After combined therapy, the number of tumors in Il1r2^−/−^ mice decreased significantly, especially in the distal colon (Fig. 1c, d, Figure S1D). We also observed similar results in male mice (Figure S1A-S1C). To evaluate the histopathological features and grade of colon tumors following combination therapy, hematoxylin and eosin (H&E) staining was performed on tumor tissue sections from wild-type and Il1r2^−/−^ mice. This analysis aimed to assess tumor differentiation, cellular morphology, and architectural patterns, which are key indicators of tumor grade and potential aggressiveness (Fig. 1f). The results indicated that tumors from the Il1r2^−/−^ mice that are treated with ICIs had significantly lower histological scores compared to other groups (Fig. 1g). These data suggest that Il1r2^−/−^ mice respond better to immune checkpoint blockade.

### Single-cell transcriptomics elucidates immunotherapy-induced alterations in the tumor microenvironment (TME)

**Fig. 2:**
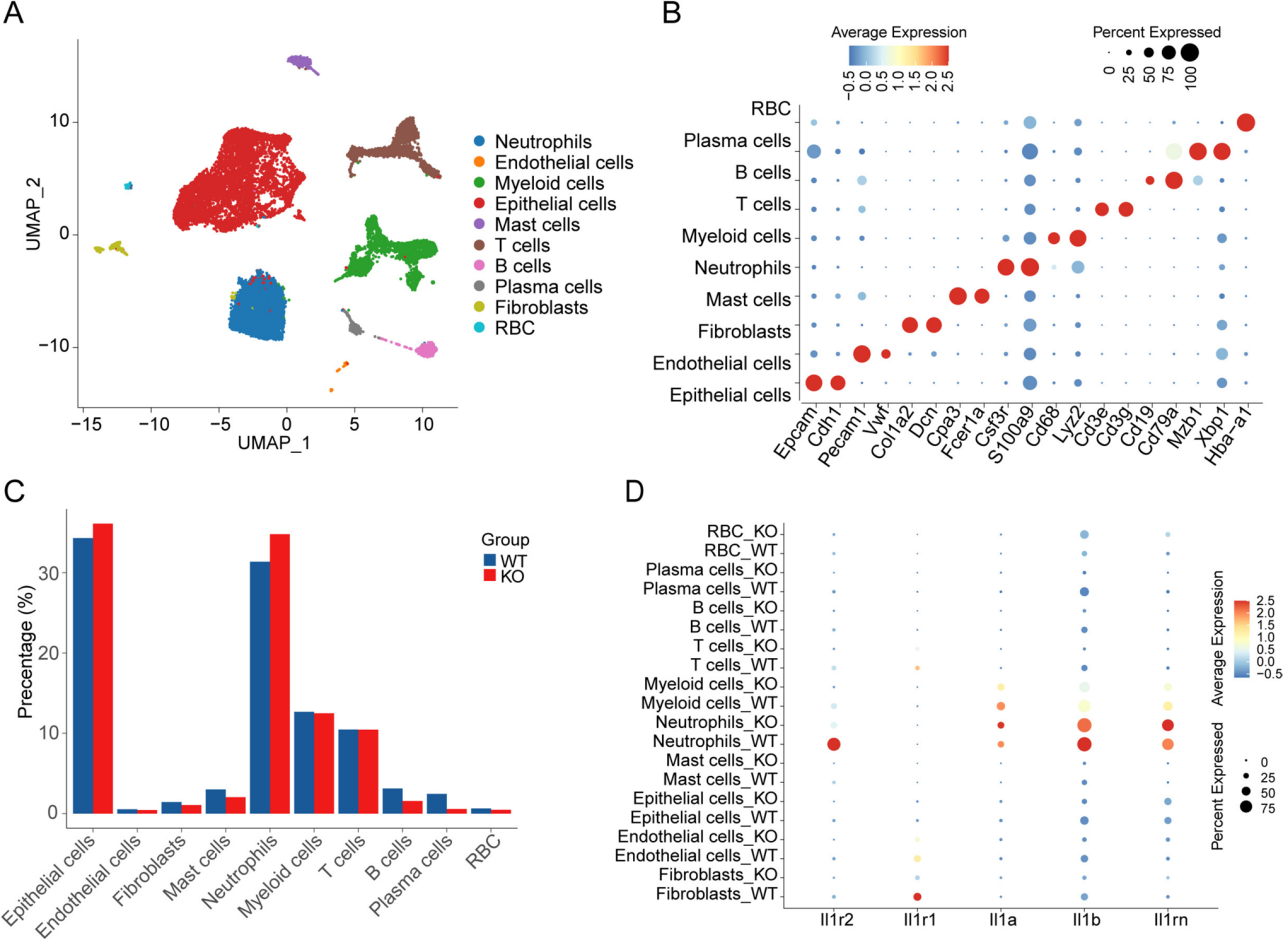
Single-cell sequencing reveals the tumor microenvironment after immunotherapy. **a** Uniform manifold approximation and projection (UMAP) plot of all 20,675 cells, colored according to cell type, three mice per group. **b** Dotplot of the expression of characteristic marker genes in the 10 identified cell clusters. **c** Barplot shows the proportion of each cluster in different treatment groups. **d** Dotplot of IL-1 related genes expressed on cell clusters

To elucidate the regulatory role of IL-1R2 in the TME and its impact on immune checkpoint blockade therapy, we conducted a comprehensive study using a CRC model. CRCs were induced in wild-type and Il1r2^−/−^ mice, followed by treatment with ICIs. Twenty-four hours after completing the combination therapy, we harvested tumor samples for analysis. To gain a high-resolution understanding of the cellular composition and transcriptional states within the TME, we performed whole-tumor single-cell RNA sequencing (scRNA-seq) on these CRC tumors. After strict quality control, we obtained a total of 20,675 cells. Unsupervised clustering analysis successfully identified ten distinct cell subpopulations, including B cells, endothelial cells, epithelial cells (both tumor and normal cells), fibroblasts, mast cells, myeloid cells, neutrophils, plasma cells, red blood cells (RBCs), and T cells (Fig. 2a and b). Tumors from Il1r2^−/−^ treated mice showed a slight increase in epithelial cell and neutrophil proportions, along with a modest decrease in B cell and plasma cell proportions (Fig. 2c). To further explore the direct effects of IL-1R2 loss on different cell populations, we examined the expression of IL-1 (including IL-1α, IL-1β) and its receptors (IL-1R1, IL-1R2) in each cell type. The results showed that *Il1a* and *Il1b* were mainly expressed in neutrophils and myeloid cells, while *Il1r1* was mainly expressed in fibroblasts, T cells, and endothelial cells. *Il1r2* was mainly expressed in neutrophils, myeloid cells, and T cells (Fig. 2d).

### IL-1R2 deletion altered oncogenic and immunogenic programs in tumor cells

**Fig. 3:**
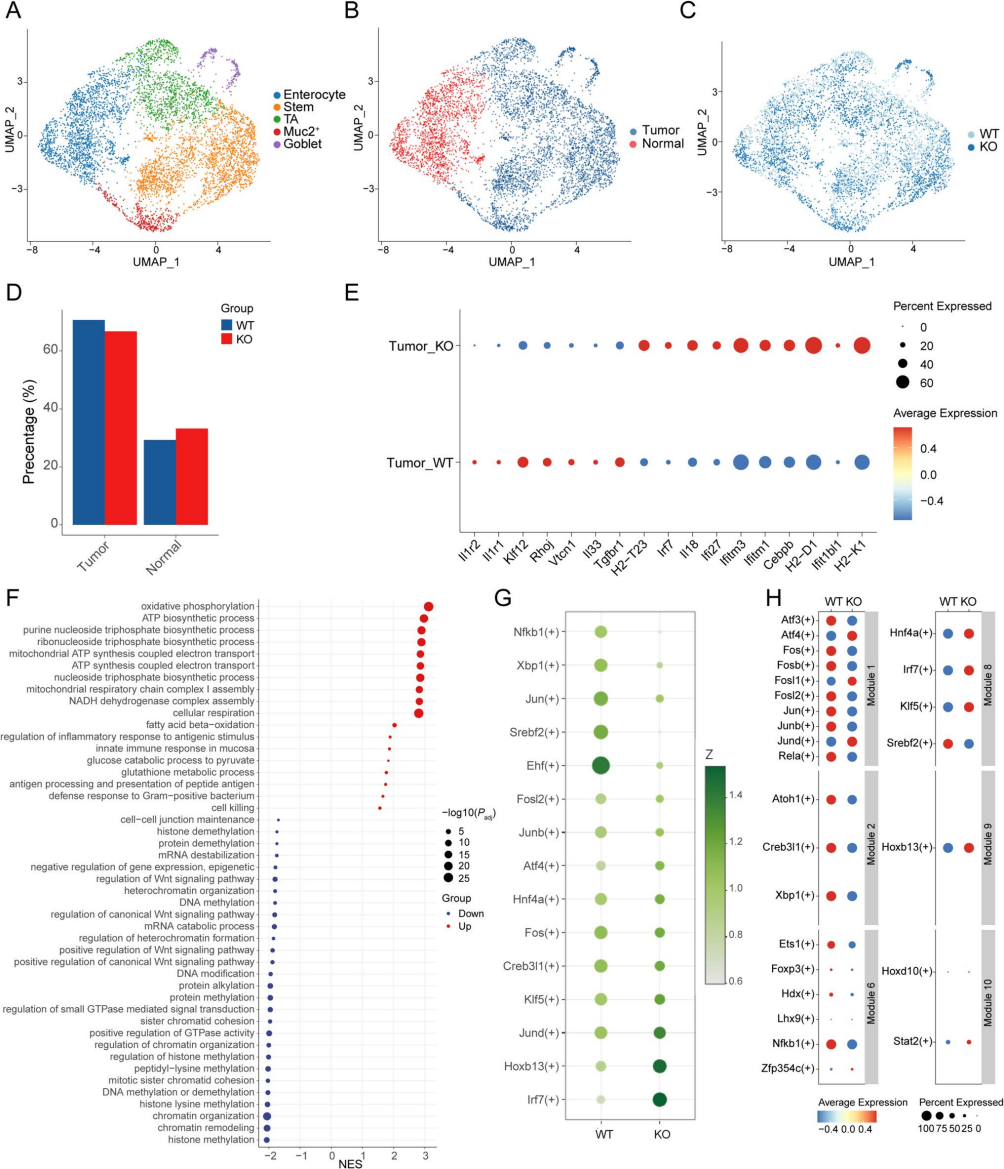
Loss of IL-1R2 reduced tumor cell proportion and function. **a** UMAP plot of epithelial cells, colored according to cell type, three mice per group. **b** UMAP of epithelial cells, colored according to cell type, three mice per group. **c** UMAP plot of epithelial cells, colored according to group, three mice per group. **d** Barplot shows the proportion of epithelial cell subsets in different treatment groups. **e** Dotplot shows differentially expressed genes in tumor cells. **f** Enrichment plot of GO terms for differentially expressed genes in tumor cells. Red represents upregulation in the KO group and blue represents upregulation in the WT group. **g** Dotplot shows specific regulons, the depth of the color represents the z-score value, and the size of the point represents the RSS score. **h** Dotplot shows the differences in regulons within tumor cells in different treatment groups

Next, we investigated the changes in epithelial cell subpopulations in the TME. 5748 epithelial cells were analyzed, and the copy number variation of single cells was calculated by the infercnv algorithm to differentiate them into normal epithelial cells and tumor cells (Fig. 3a–c, Figure S2A). The results showed that the proportion of epithelial cell subsets changed little after IL-1R2 deletion (Fig. 3d). We analyzed the expression of IL-1 signal-related genes in epithelial cells, and the results showed that the expression of *Il1r1* was decreased in tumor cells after the deletion of IL-1R2, suggesting a decreased IL-1 signaling in Il1r2^−/−^ tumor cells. Further analysis of differentially expressed genes in tumor epithelial cells showed that tumor cells in Il1r2^−/−^ mice showed increased expression of *Il18* (Fig. 3e), and IL-18 is known to have antitumor properties^17^, ^18^. At the same time, the expression of MHC class I-related molecules (*H2-D1, H2-K1, H2-T23*) and the immune response-related genes *Irf7*, *Ifi27* was increased in Il1r2^−/−^ mouse tumor cells. Genes associated with cell migration and proliferation, such as *Klf12*, *Rhoj*, and *Vctn1*, were down-regulated in IL-1R2 deficient tumor cells. These data are consistent with the idea that Il1r2 deficiency led to an increase in immunogenicity of tumor cells.

GSEA results showed that up-regulated genes in Il1r2^−/−^ tumor cells were enriched in energy metabolism and antioxidant-related processes such as oxidative phosphorylation, ATP synthesis, nucleoside triphosphate biosynthetic process, and glutathione metabolism, indicating high energy requirements and enhanced antioxidant capacity. Immune-related pathways such as antigen processing and presentation of peptide antigen and innate immune response in the mucosa were also enriched in Il1r2^−/−^ tumor cells. Down-regulated pathways in Il1r2^−/−^ tumor cells included histone demethylation, negative regulation of gene expression, cell junction maintenance, and Wnt signaling pathway that may lead to genomic instability and enhanced invasiveness. In addition, the regulatory imbalance of the Wnt signaling pathway further supports the abnormal proliferation and differentiation of WT tumor cells (Fig. 3f). The enrichment analysis results of Kyoto encyclopedia of genes and genomes (KEGG) and REACTOME also showed similar results (Figure S2B-S2C).

In order to reveal the regulation mechanism of gene expression at the level of a single cell, we conducted a single-cell regulatory network Inference and clustering (SCENIC) analysis on tumor cells. It helps us to understand which key transcription factors are regulating gene expression and, at the same time, allows us to assess the activity of these discovered regulons (i.e., transcription factors and their target genes) in individual cells, leading to a better understanding of the functional state of the cell. The Regulon Specificity Score (RSS) quantifies the activity and specificity of transcriptional regulons within a cell population, with higher values indicating increased activity or specificity. Analysis of RSS values revealed distinct regulatory patterns between WT and Il1r2^−/−^ tumor cells. In WT tumor cells, regulons associated with NFKB1, XBP1, JUN, SREBF2, and EHF exhibited elevated activity. Conversely, Il1r2^−/−^ tumor cells demonstrated higher specificity for regulons controlled by IRF7, HOXB13, and JUND. These differential regulatory profiles suggest distinct transcriptional programs governing the behavior of WT and Il1r2^−/−^ tumor cells, potentially contributing to their varied responses to therapy (Fig. 3g, Figure S2D).

To further establish modularity and structure of the transcription factor network, we used the connection specificity index (CSI) method for module analysis. We performed CSI calculations based on AUC values for each regulon to quantify the specificity of these regulatory relationships under different conditions. Regulons with higher CSI values may have similar cellular functions and co-regulate downstream genes. Regulons are divided into ten major modules, each containing one or more transcription factors and their potential target genes (Figure S2E). Module 1 contains ATF3, FOS, FOSB, JUN, JUNB, and other regulons, most of which belong to the activator protein-1 (AP-1) complex family members, mainly involved in the regulation of stress response, cell proliferation, differentiation, and apoptosis pathways. It was also down-regulated in Il1r2^−/−^ tumor cells. Module 2 contains ATOH1, CREB3L1, XBP1, and other regulons related to ER stress response and unfolded protein response (UPR). Module 3 is a highly diverse module involved in immune regulation (e.g., IRF5, IRF8), cell differentiation (e.g., CEBPB, RUNX3, SPI1), and epithelial-mesenchymal transition (ZEB1) regulation. Module 4 contains regulons related to cell cycle regulation and neural development, such as E2F1, GLI3, and NEUROD1. Modules 5, 6, and 7 are closely related to cell differentiation and development. Module 8 was implicated in metabolic regulation and immune responses, such as HNF4A and IRF7, and was upregulated in Il1r2^−/−^ tumor cells. Modules 9 and 10 include HOXB13 and HOXD10, both of which belong to the HOX gene family and are associated with cell migration and invasive capacity, and both transcription factors showed upregulation in Il1r2^−/−^ tumor cells. Meanwhile, STAT2, a key molecule in the JAK-STAT signaling pathway, also showed upregulation in Il1r2^−/−^ tumor cells (Fig. 3h).

Taken together, IL-1R2 deletion resulted in significant down-regulation of transcription factor modules related to key biological processes such as stress response, immune regulation, cell differentiation, and development.

### IL-1R2 deletion led to an increase of exhausted CD8^+^ T cells in the TME

**Fig. 4:**
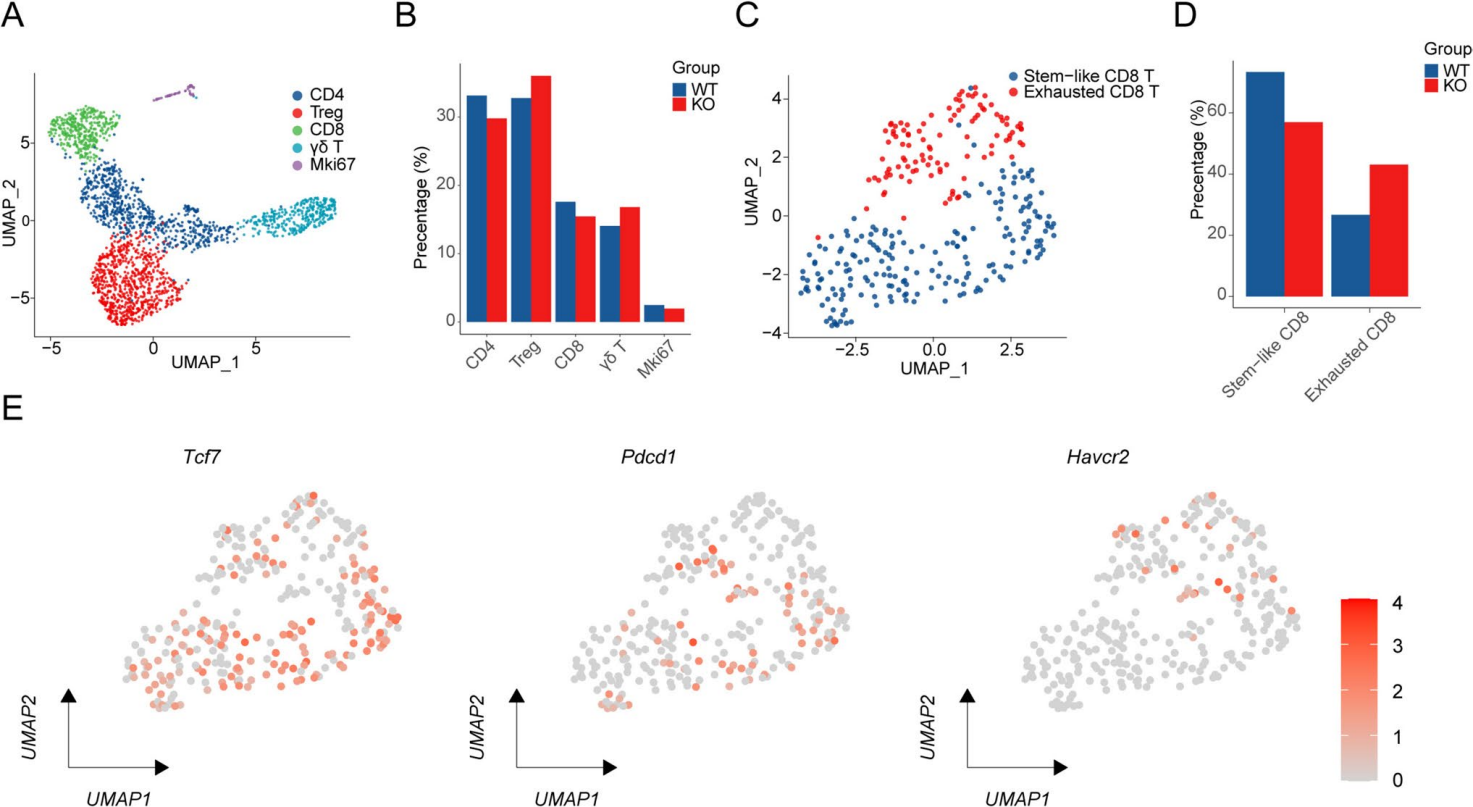
Depletion of IL-1R2 promotes Exhausted CD8^+^ T cell infiltration and function. **a** UMAP plot of T cells, colored according to cell type, three mice per group. **b** Barplot shows the proportion of each T cell subset in different treatment groups. **c** UMAP of CD8^+^ T cells from all samples, colored according to cell type, three mice per group. **d** Barplot shows the proportion of CD8⁺ T cell subsets in different treatment groups. **e** Feature plot shows the expression of representative genes (e.g., Tcf7, Pdcd1, Havcr2) across CD8^+^ T cell

We further evaluated T cell subsets, which were subdivided into five subsets, including CD4^+^ T cells, Treg cells, CD8^+^ T cells, γδ T cells, and MKi67-expressing T cells (Fig. 4a). The analysis showed a slight decrease in the proportion of CD4^+^ T cells and CD8^+^ T cells and a slight increase in Treg cells in Il1r2^−/−^ TME (Fig. 4b). To gain deeper insights into CD8^+^ T cell functionality, we further categorized α/β CD8^+^ T cells into two distinct subsets: stem-like CD8^+^ T cells (Tcf7, Sell, Lef1) and exhausted CD8^+^ T cells (Pdcd1, Havcr2, Lag3) (Fig. 4c and e). The results showed that IL-1R2 deficiency led to an increase in the proportion of exhausted CD8^+^ T cells (Fig. 4d). Further analysis revealed that these cells also expressed certain levels of the cytotoxic-related gene Prf1. In recent studies, this type of ‘functionally preserved exhausted T cell’ has been identified as an important subgroup with residual killing capacity in tumor immunity. Despite their nomenclature, exhausted T cells are often enriched with tumor-specific T cells and can serve as surrogate indicators of antitumor immune responses^19^, ^20^. This paradoxical relationship arises from the persistent antigen exposure and immunosuppressive tumor microenvironment, which drive T cell exhaustion while simultaneously selecting for tumor-reactive clones. Therefore, our data suggest an enhanced antitumor CD8^+^ T cell immune response in Il1r2^−/−^ tumors.

### IL-1R2 deletion remodeled Treg cell function

**Fig. 5:**
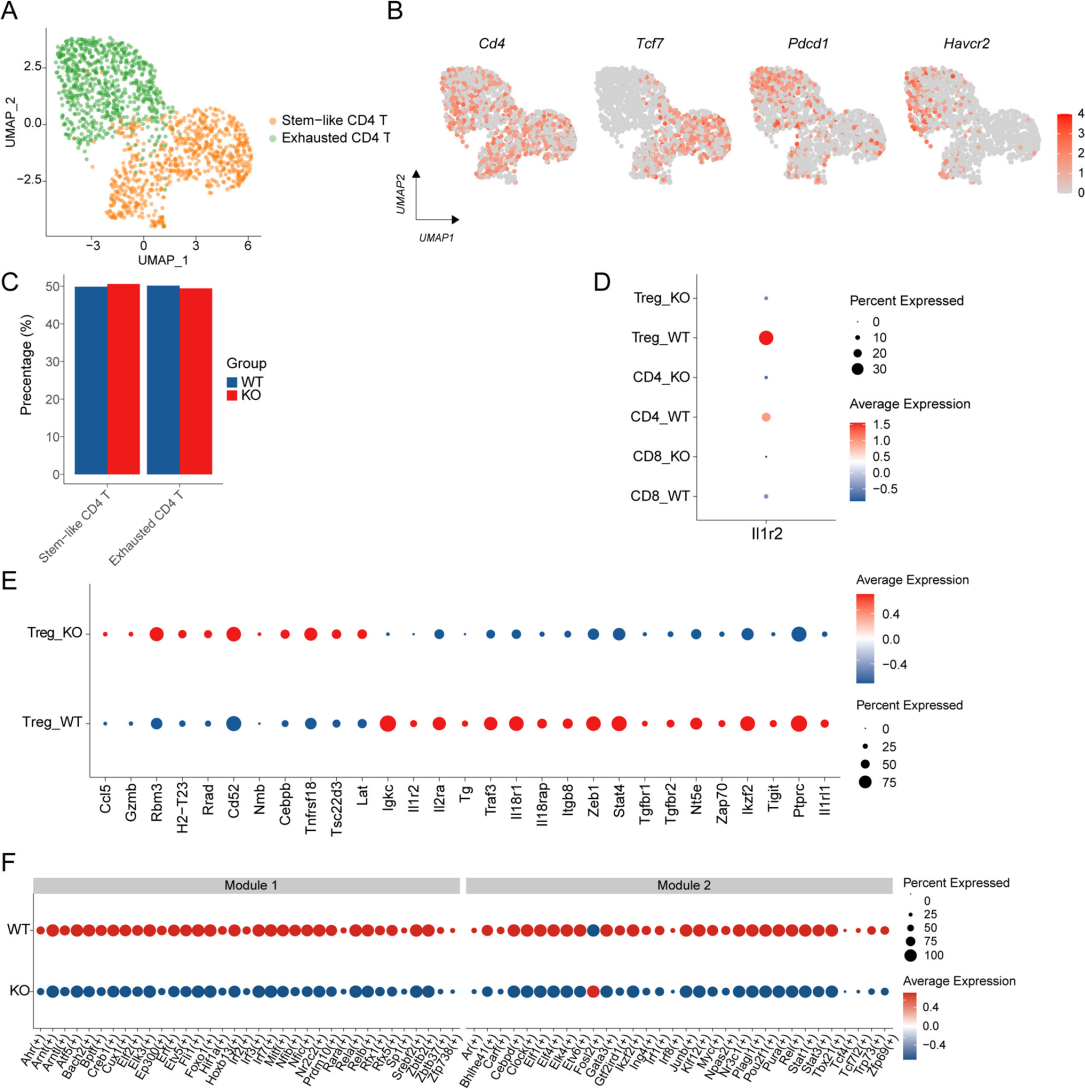
IL-1R2 depletion remodels Treg cell function. **a** UMAP of CD4^+^ T cells, colored according to cell type, three mice per group. **b** UMAP figure shows the characteristics of CD4^+^ T cell gene expression. **c** Barplot shows the proportion of CD4^+^ T cell subsets in different treatment groups. **d** Dotplot shows the expression of IL-1R2 on T cell subsets. **e** Dotplot shows functional genes of Treg cell subsets. **f** Dotplot shows he difference regulons in Treg cell in different treatment groups

Subsequently, we carried out a detailed analysis of CD4^+^ T cells and divided them into stem-like CD4^+^ T cells and exhausted CD4^+^ T cells (Fig. 5a). UMAP showed expression patterns of stem-like and exhausted marker genes (Fig. 5b). In addition, we compared changes in the proportion of CD4^+^ T cell subsets and found that no significant difference was observed between the IL-1R2 deficient and control groups (Fig. 5c).

Treg cells maintain immune system balance through various inhibitory mechanisms, thereby influencing inflammatory responses and tumor progression^21^, ^22^. Studies have shown that infiltration of eTreg (Treg subgroup with highly inhibitory activity) in patients with CRC is often associated with poor prognosis^23^, ^24^. Therefore, reducing or depleting Treg cells is seen as an effective strategy to activate an anti-tumor immune response. Single-cell sequencing revealed high expression of IL-1R2 on Treg cells (Fig. 5d). In order to determine the effect of IL-1R2 loss on Treg cell function, we conducted an in-depth analysis of genes related to the function and regulation of Treg cells (Fig. 5e). We found that the expression of signaling molecule *Zap70*, IL-2 signaling pathway-related gene *Il2ra* (CD25), immunosuppressive molecule *Tigit*, and immune regulatory factors *Ikzf2* (also called Helios), *Nt5e* (also called CD73), and *Ptprc* were significantly down-regulated in Il1r2^−/−^ Treg cells. In addition, the expression of TGF-β receptors on the surface of Treg cells, including *Tgfbr1* and *Tgfbr2*, was significantly reduced in Il1r2^−/−^ Treg cells. These results suggest that IL-1R2 deficiency leads to reduced immunoregulatory and inhibitory functions of Treg cells. At the same time, we also found that IL-1R2 deletion resulted in decreased expression of *Il1rl1* on Treg cells, suggesting that its role in promoting tumor development may be reduced^25^. In contrast, the expression of the signaling molecule *Lat* was upregulated. Genes related to cell survival and anti-inflammatory, such as *Rbm3*, *Rrad*, *Tnfrsf18*(GITR), *Tsc22d3* (GILZ), and *Cebpb,* were up-regulated in Il1r2^−/−^ Treg cells. These findings reveal the potential regulatory role of IL-1R2 on Treg cell function. Next, SCENIC technology was used to analyze transcription factor changes in Treg cells after IL-1R2 deletion. The results showed that transcription factors such as FOXO1, PLAGL1, ELK3, NFIB, STAT1, and IKZF2 were significantly down-regulated in Il1r2^−/−^ Treg cells (Figure S3A). To further study how transcription factor coordinates gene expression, we analyzed the significant correlation between different regulators based on CSI and identified eight regulon modules (Figure S3B). We found that regulon such as AHR, IRF3, IRF7, BACH2, RELA, RELB, FOXO1, HIF1A, and SREBF2 are all clustered in Module 1, and these factors have been reported to be related to immune and metabolic regulation.

However, in Module 2, several regulons related to immune regulation and inflammatory response, such as FOSL2, GATA3, IKZF2, JUNB, STAT1, REL, as well as regulons related to cell growth, proliferation, and metabolism, such as MYC, CEBPD, KLF12, NR3C1, and AR have been identified. These regulons were significantly down-regulated in Il1r2^−/−^ Treg cells, suggesting that gene knockout may impair the immunomodulatory function of Treg cells, thereby reducing their ability to regulate immune balance, and may have an impact on the overall immune response (Fig. 5f).

### Loss of IL-1R2 promotes the expression of antigen-presenting genes on DC cells.

**Fig. 6:**
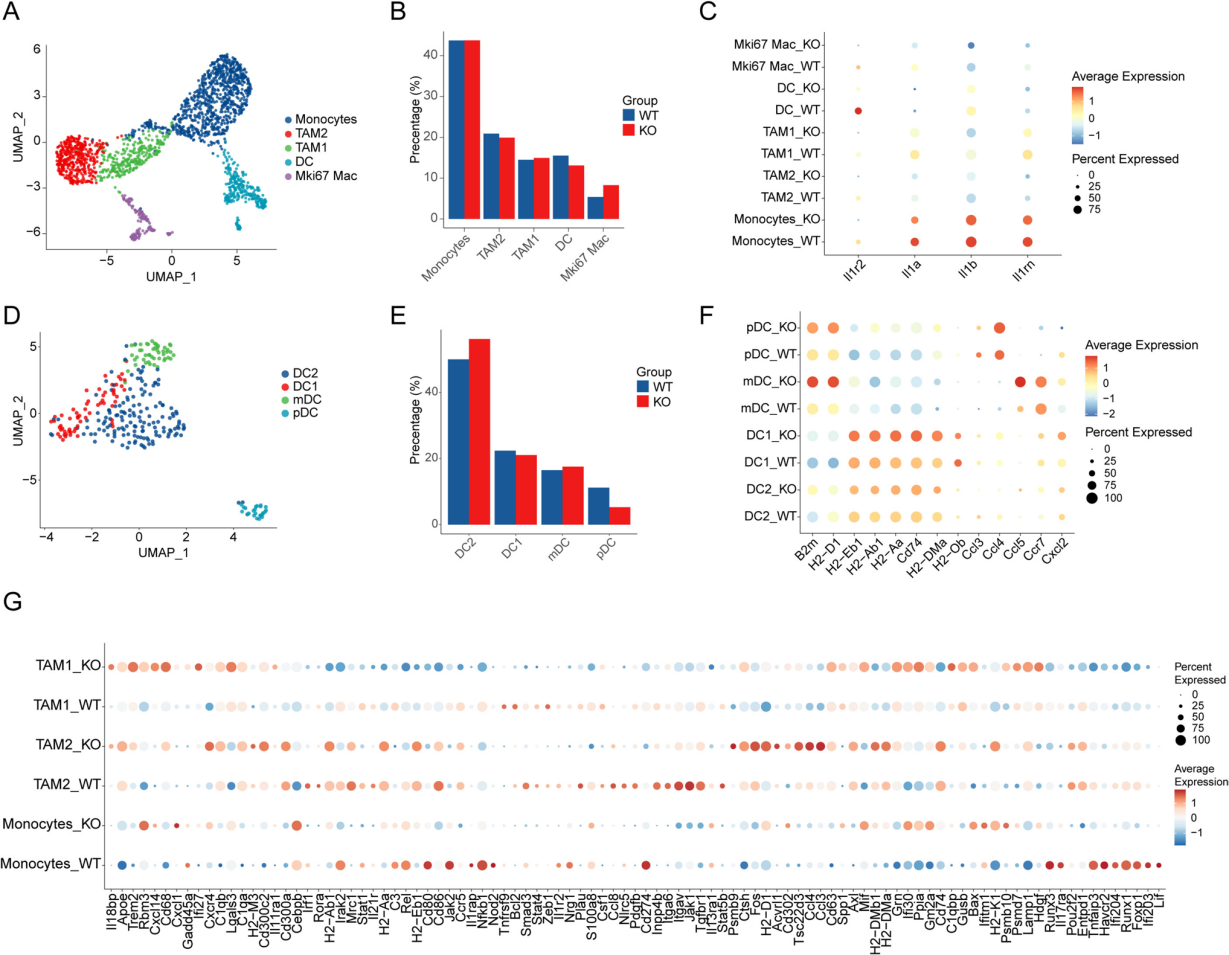
Loss of IL-1R2 promotes the expression of antigen-presenting genes on DC cells. **a** The myeloid cells of the UMAP figure, according to the cell type coloring, three mice in each group. **b** Barplot shows the proportion of each cluster in different treatment groups. **c** Dotplot shows the expression of IL-1-related genes on cell clusters. **d** UMAP plots of DC cell subsets, colored according to cell type. **e** Barplot shows different percentages of each cluster in the treatment group. **f** Dotplot shows functional genes of DC cell subsets in different treatment groups. **g** Dotplot shows differentially expressed genes in myeloid cell subsets in different treatment groups

In 1965, myeloid cell samples, we successfully identified five subpopulations by cluster analysis, including monocytes, tumor-associated macrophage 1 (TAM1), tumor-associated macrophage 2(TAM2), Dendritic cells (DCs), and Mki67 macrophage (Fig. 6a, Figure S4A). Cell proportion analysis showed that there was no significant difference in cell distribution between the two groups, although DC cells were somewhat reduced (Fig. 6b). Considering that IL-1R2 is expressed in myeloid cells, we performed an exhaustive analysis of its expression in each myeloid cell subset. The results showed that *Il1r2* was mainly expressed in DC cells, while *Il1a*, *Il1b*, and *Il1rn* were highly expressed in monocytes (Fig. 6c). To further elucidate the composition of DCs in our model, we conducted a comprehensive analysis of the DC population. Based on the expression profiles of characteristic marker genes, we delineated four distinct DC subsets: DC1, DC2, myeloid DC (mDC), and plasmacytoid DC (pDC) (Fig. 6d, Figure S4B). Quantitative analysis of these DC subsets revealed significant alterations in their proportions following IL-1R2 knockout. Notably, DC2 cells exhibited a slight increase in proportion, while pDC cells showed a notable decrease. In contrast, the relative abundances of DC1 and mDC subsets remained largely unchanged (Fig. 6e). Considering that DC cells, as one of the major antigen-presenting cells, play a key role in activating T cells and regulating the body's immune response^26^, ^27^, we looked at the expression of antigen-presentation-related genes and chemokines. After the loss of IL-1R2, MHC class I molecules (e.g., *H2-D1*, *B2m*) in mDC, pDC, and DC1 cells expression on an increase. Meanwhile, the expression of MHC class II-related molecules (*H2-Eb1*, *H2-Ab1*, *H2-Aa*, *H2-DMa*, and *Cd74*) was also increased on DC1 cells. Chemokines CCL4 and CCL5 play an important role in DC-induced T cell migration and activation^28^, ^29^, and their expression was up-regulated in Il1r2^−/−^ DC cells. CCR7 was mainly expressed on DC cells, which were involved in the migration of DC to lymph nodes and T cells^30^, ^31^. The expression of *Ccr7* on mDC cells was also increased compared with the WT group (Fig. 6f). These results suggest that IL-1R2 deletion enhances the antigen presentation capacity of DC cells, which contributes to the activation of T cells and elicits specific immune responses.

Differential gene analysis of macrophages showed that the expression of genes related to inflammation, such as *Il18bp*, *Apoe*, *Trem2,* and *Mif* in TAM1, was up-regulated after IL-1R2 deletion. Genes related to cellular stress response, such as *Gadd45a* and *Ifi27*, were also upregulated in TAM1. In addition, the up-regulation of *C1qb*, *C1qa*, *Lamp1*, *Lgals3*, *Gm2a*, *Gusb*, *Bax,* and other genes helps to enhance the anti-tumor and anti-infection ability of the immune system. The expression of *Psmb9*, *Ctsh*, *Fos*, *H2-D1*, *Acvrl1*, *Cd302*, *Tsc22d3*, *Ccl4*, *Ccl3* and *Cd63* genes in TAM2 was up-regulated after IL-1R2 deletion. Upregulation of these genes is often associated with enhanced tissue repair, anti-inflammatory response, and immunomodulatory function. After IL-1R2 loss, the expression of *Runx3*, *Il17ra*, *Nfkb1*, *Il1rap*, *Jak2*, and *Lif* on monocytes is down-regulated, which may reduce the inflammatory response and cell activation capacity of monocytes, while the down-regulation of *Cd80*, *Cd86*, and *Ccr5* may affect their roles in antigen presentation and T cell activation (Fig. 6g).

We analyzed the differentially expressed genes in neutrophils and found that the expression levels of *Osm*, *Hsd11b1*, *Slc15a2*, and *Dmbt1* were decreased in mice with IL-1R2 deletion, while the expressions of *Ccrl2*, *Cxcr4*, *Icam1*, *Ifi27l2a*, and *Dusp2* were up-regulated (Figure S5A). GO pathway enrichment analysis showed that aerobic electron transport chain function was up-regulated in Il1r2^−/−^ mice, suggesting that neutrophils may promote immune response by enhancing oxidative phosphorylation or production of reactive oxygen species (ROS). At the same time, complement activation, phagocytosis recognition, wound healing, and cell junction assembly were down-regulated. These results suggest that the immune and tissue repair functions of neutrophils are reshaped dynamically (Figure S5B). Reactome pathway enrichment analysis showed that neutrophil metabolism and effector functions (such as amino acid metabolism, translation, and antigen processing) were significantly enhanced in the activated state. At the same time, activities related to heme clearance, intercellular communication, and migration are inhibited, reflecting dynamic changes in its function in a given environment (Figure S5C).

### IL-1R2 deletion modulates fibroblast subpopulation dynamics and IL-1 signaling

The previous results showed that *Il1r1* was highly expressed on fibroblasts (Fig. 2d).

**Fig. 7:**
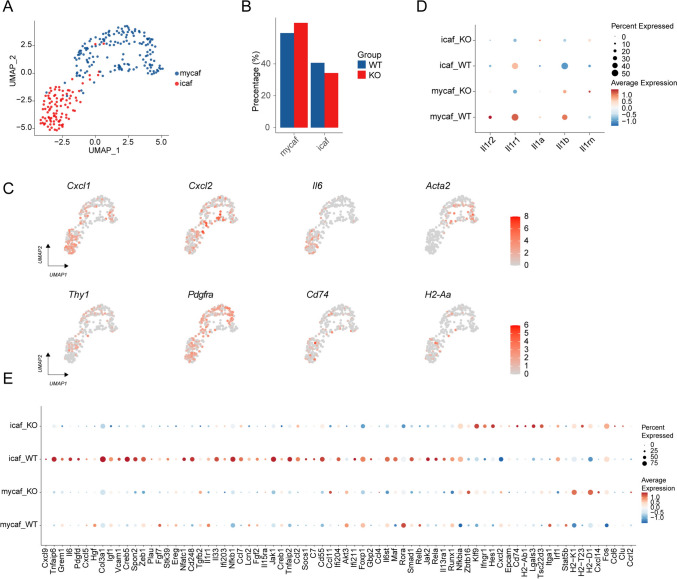
IL-1R2 deletion modulates fibroblast subpopulation dynamics and IL-1 signaling. **a** Fibroblasts of the UMAP figure, according to the cell type coloring, three mice in each group. **b** Barplot shows the proportion of each cluster in different treatment groups. **c** UMAP map showing characteristic genes of fibroblasts. **d** Dotplot showing the expression of IL-1 related genes on cell clusters. **e** Dotplot shows differentially expressed genes in fibroblast subsets in different treatment groups

We successfully identified two subpopulations of fibroblasts, including inflammatory fibroblasts (iCAF) and myofibroblasts (myCAF) (Fig. 7a), through the expression of characteristic genes. Analysis of cell proportions revealed that in the absence of IL-1R2, the proportion of iCAF and myCAF did not significantly change (Fig. 7b). Gene expression profile showed that myCAF mainly expressed *Acta2*, while iCAF highly expressed *Il6*, *Cxcl1,* and *Cxcl2* (Fig. 7c). To investigate the effect of IL-1R2 loss on IL-1 signaling in cell subsets, we analyzed the expression of genes associated with IL-1 signaling. The results showed that the expression of *Il1r1* was significantly down-regulated after the absence of IL-1R2 (Fig. 7d). In addition, we compared the expression differences between different subpopulations and identified a series of differentially expressed genes associated with specific functions (Fig. 7e). The deletion of IL-1R2 led to the downregulation of *Cxcl9*, *Il6*, *Ccl2*, *Ccl4*, *Ccl7,* and other inflammatory response-related genes in iCAF cells, as well as the downregulation of *Jak1*, *Jak2*, *Akt3*, *Creb1*, *Creb5,* and other genes in the PI3K-Akt signaling pathway. In addition, genes such as *Col3a1*, *Zeb1*, and *Plau*, which are related to matrix remodeling and migration, were also down-regulated. The up-regulation of *Ifngr1* expression in Il1r2^−/−^ iCAF can potentially help enhance the response to interferon-gamma and promote anti-tumor immunity. At the same time, IL-1R2 deletion also led to up-regulation of *H2-Ab1*, *H2-K1*, *H2-T23*, *H2-D1*, and other antigen-presenting genes on iCAFs. *Epcam*, *Itga1*, *Lgals3,* and other genes involved in intercellular adhesion were also significantly up-regulated after IL-1R2 deletion in iCAF. These findings suggest that IL-1R2 might affect the TME via its action on CAFs.

## Discussion

Our study reveals a previously unrecognized role for IL-1R2 in modulating immune responses in CRC. Using an AOM/DSS-induced colitis-associated CRC model, we demonstrated that IL-1R2 deletion significantly reduces tumor burden. The reduction of tumor burden is more significant when combining IL-1R2 deletion and ICIs. Notably, the deletion of IL-1R2 was associated with increased CD8^+^ T cell exhaustion and enhanced antigen presentation by dendritic cells, suggesting that IL-1R2 modulates both the immunogenicity of tumor cells and the functionality of key immune populations within the TME. These findings provide a strong rationale for further exploration of IL-1R2 as a therapeutic target in combination with immune therapies, with the potential to overcome resistance and broaden the scope of immunotherapy in CRC.

ICI has been approved for metastatic MSI-H CRC. In a Phase II non-comparative study, patients treated with nivolumab in combination with ipilimumab demonstrated notable clinical benefits, with an objective response rate of 71%^32^. In non-metastatic colon cancer patients treated with a combination of anti-CTLA-4 and anti-PD-1 monoclonal antibodies, the pathological response rate of dMMR tumors reached 100%, and only 13% of patients experienced immune-related adverse reactions, suggesting that this is a safe and effective treatment strategy^33^. A follow-up study shows that in patients with nonmetastatic locally advanced dMMR colon cancer, neoadjuvant nivolumab plus ipilimumab demonstrated outstanding efficacy and a favorable safety profile. A pathological response was observed in 98% of patients, with 68% achieving a pathological complete response. Additionally, treatment was well tolerated, with 98% of patients undergoing surgery as planned and only 4% experiencing grade 3 or 4 immune-related adverse events^34^. However, anti-PD-1 monotherapy has not demonstrated clinical benefit in patients with pMMR CRC and is not considered standard of care for this population. In contrast, recent clinical data have shown that the combination of anti-CTLA-4 and anti-PD-1 yields promising clinical responses in pMMR CRC patients without liver metastases. In the response-evaluable population, the objective response rate (ORR) of patients was 17%^35^. These findings suggest that this combination has great potential to become a standard of care for pMMR CRC without liver metastasis. This is the primary rationale for selecting the anti-CTLA-4 and anti-PD-1 combination in our study—to maintain clinical relevance. Single-cell data showed that the high expression of IL-1R2 on tumor-infiltrating Treg cells^13^ and the loss of IL-1R2 helped to increase the infiltration of CD8^+^ T cells in TME, and the anti-tumor effect was significant 14. Therefore, the combination of IL-1R2 blocking and immune checkpoint inhibitors is expected to enhance the body's immune system to kill tumors. Our results showed that fewer tumors were observed in Il1r2^−/−^ mice after combination treatment with anti-CTLA-4 and anti-PD-1 monoclonal antibodies. Histopathological analysis showed that the tumor malignancy of Il1r2^−/−^ mice after treatment was significantly reduced compared with the control group, which further indicated that IL-1R2 blocking combined with immune checkpoint inhibitor therapy can effectively slow tumor growth. In fact, single-cell sequencing results showed that fewer tumor cells were observed in Il1r2^−/−^ mice, suggesting that IL-1R2 deletion may lead to reduced tumor epithelial cell proliferation.

IL-1 family molecules exhibit complex roles in inflammatory diseases and tumorigenesis. IL-1α plays a dominant role in the pathogenesis of DSS-induced acute colitis, and intestinal epithelium-specific knockout of IL-1α significantly improved the symptoms of colitis, underscoring the critical proinflammatory role of epithelium-derived IL-1α. IL-1β null mice, however, showed increased inflammation during acute injury and a weaker effect during the repair phase, with still significant inflammatory cell infiltration and lower expression of PCNA and the crypt tight junction protein Claudin-3 than wild-type mice^36^. There was no significant difference in the severity of colitis between mice with IL-1R1 deletion and the wild-type control group^37^. These findings reflect the complex roles of IL-1 in colitis, likely due to their pleiotropic functions. For example, IL-1 signaling on fibroblasts has been shown to promote wound healing, and IL-1 signaling on myeloid cells might be proinflammatory^38^, ^39^. IL-1R2 has also been studied in the ulcerative colitis model using DSS; the degree of inflammation in mice with IL-1R2 deficiency is reduced, suggesting that IL-1R2 plays a pro-inflammatory role in this model^37^. This finding is consistent with our data, which further emphasizes the contribution of IL-1R2 to the inflammatory process. These findings suggest the diverse role of IL-1 family members. However, the lack of precise deletion of these molecules in various cell types limits the generalization of these findings.

Consistent with findings in colitis, IL-1α-deficient mice in the AOM/DSS-induced model experienced less weight loss and had lower pathological scores compared to WT mice. Moreover, IL-1α-deficient mice significantly suppressed tumor growth in the context of IL-33 deletion-induced colitis, highlighting IL-1α’s crucial role in promoting colitis and tumor development^40^. In the colitis-associated cancer model, IL-1R antagonist (IL-1Ra) treatment results in decreased tumor formation^41^. However, it has been shown that IL-1β-deficiency does not affect tumor formation in the AOM/DSS model^42^. Interestingly, there was no significant difference in the number of tumors in Il1r1^−/−^ mice compared to wild-type mice in AOM/DSS-induced colon cancer models^18^. However, in the CPC-APC (CDX2^Cre^-Apc^f/wt^) colorectal cancer model, specific deletion of IL-1R1 in myeloid cells (CD11b^Cre^-Il1r1^f/f^) resulted in a higher tumor load and stronger inflammatory responses, such as increased IL-17A expression^43^. These findings underscore the complex roles of IL-1α and IL-1β in CRC tumorigenesis, likely due to their distinct functions in different target cells, which warrant further investigation. Our data suggest that blockade of IL-1R2 reduces inflammation and tumor progression. However, the exact role of IL-1R2 on each type of cell needs to be further studied.

Our scRNA-seq analysis suggests IL-1R2 affects tumorigenesis through multiple mechanisms. In tumor cells, we found that IL-1R2 deficiency led to decreased tumorigenesis and increased immunogenic programs. Intracellular IL-1R2 may function to promote tumorigenesis. Consistent with this notion, it was found that intracellular IL-1R2 enhanced the proliferation and migration of colon cancer cells by mediating the expression of cytokines, including VEGF and IL-6^15^. IL-1β can promote the release of the intracellular domain of IL-1R2 (icd-IL-1R2), and the increased IL-1R2 in breast cancer can promote the self-renewal and proliferation of breast tumor cells^16^. It is, however, also possible, tumor-extrinsic mechanisms also contribute to the reduced tumorigenesis.

Exhausted CD8^+^ T cells are thought to be antigen-specific, have a high expression of PD-1, and are believed to have powerful anti-tumor functions. The presence of CD8^+^ PD-1^+^ T cells is also the only biomarker that predicts the immune response generated by pMMR tumors 33. In this study, we found that after the absence of IL-1R2, the number of Exhausted CD8^+^ T cells increased, which suggested that IL-1R2 had a certain regulatory effect on CD8^+^ T cells.

IL-1R2 is highly expressed in DC cells. The analysis results of functional genes related to DC cells showed that the expression of antigen presenting genes (such as *H2-D1*, *B2m*, *H2-Eb1*, *H2-Ab1*, *H2-Aa*, *H2-DMa* and *Cd74*) was up-regulated, and the expressions of chemokines that promote the migration of DC cells and T cells, such as CCL4, CCL5 and CCR7, were also up-regulated. This will help DC cells to present antigens to CD8^+^ T cells and promote the effector function of CD8^+^ T cells, consistent with increased levels of exhausted CD8^+^ T cells in IL-1R2 deficient tumors.

Treg cells, as immunosuppressive cells in TME, can promote tumor occurrence^44^. Recent studies have found that Treg cells with high expression of IL-1R1 have enhanced immunosuppressive ability^45^. When IL-1R1 is absent on T cells, tumor growth and progression of CRC are inhibited^43^. In this study, the absence of IL-1R2 resulted in significantly reduced IL-1R1 expression on Treg cells. In addition, tumor-infiltrating Treg cells express a small amount of *Il2ra*(CD25), a TCR-mediated activation marker^46^, which competitively binds IL-2, thereby inhibiting the activity of other effector T cells and exerting immunosuppressive function. Down-regulated expression of inhibitory molecules *Tigit*, *Ikzf2*(Helios), *Nt5e*(CD73), *Tgfbr1,* and *Tgfbr2* were associated with reduced immunosuppressive ability. These findings suggest that IL-1R2 loss reduces Treg cell function, which in turn affects its ability to establish an immunosuppressive environment at the tumor site. Considering that intracellular IL-1R2 plays an important regulatory role, the importance of icd-IL-1R2 self-conduction in Treg cells cannot be a possible mechanism, along with extrinsic effects where IL-1R2 acts as an inhibitory molecule of IL-1 signaling as a decoy receptor^14^.

Interleukin-6 (IL-6) is a key pro-inflammatory cytokine that plays a central role in shaping the tumor microenvironment and has been associated with the prognosis of various cancers, including skin cancer^47^, ^48^. In our study, IL-6 expression was found to be downregulated in inflammatory cancer-associated fibroblasts (iCAFs) from IL-1R2 knockout mice. Given IL-6’s well-established roles in promoting tumor progression, angiogenesis, and immunosuppression^48^, ^49^, these findings suggest that IL-1R2 may contribute to tumor progression through its regulation of IL-6 expression in CAFs. However, further investigation is required to validate this mechanism and to elucidate the broader implications of IL-1R2 signaling in the tumor microenvironment.

IL-1R2 plays a crucial role in the tumor microenvironment, but there are currently no approved therapeutic agents targeting IL-1R2. However, studies have explored the feasibility of using neutralizing antibodies and RNA interference technology to inhibit IL-1R2 in tumor models. shRNA targeting IL-1R2 has been shown to inhibit tumor cell proliferation in osteosarcoma (U-2 OS) and colon cancer (HT29, SW620) models^15^, ^50^. Additionally, the combination of IL-1R2 neutralizing antibodies with anti-PD-1 therapy has demonstrated potent antitumor efficacy in triple-negative breast cancer (TNBC) patients^51^. These findings provide robust mechanistic evidence supporting IL-1R2-targeted interventions.

Although this study provides valuable insights, there are certain limitations: due to the use of IL-1R2 total knockout mice, it was not possible to determine which cell type plays a dominant role in the anti-tumor response. The phenotype observed in IL‑1R2–deficient mice may arise from a combination of immune‑mediated effects and intrinsic epithelial cell alterations. Therefore, in future studies, it will be key to conditionally knock out IL-1R2 in specific high-expression cells in order to provide a theoretical basis for targeted intervention. The AOM/DSS-induced model may introduce false positives in immune responses due to its inherent biological variability. In the future, it will be necessary to combine other models to more accurately investigate the role of IL-1R2 in the tumor microenvironment. In addition, the conclusion of this study is mainly based on scRNA-seq data, so the key findings will be further verified at the protein level in the future to enhance the biological basis of the proposed mechanism model and its potential clinical transformation value.

In summary, this study suggests that IL-1R2 deletion, in combination with immune checkpoint blockade therapy, further enhances therapeutic efficacy. Our data, aligning with other published data^7^, support a new strategy for leveraging IL-1R2 inhibition in combination with ICIs for colon cancer immunotherapy.

## Materials and methods

### Experimental mice

C57BL/6 J mice (6–8 weeks old) and C57BL/6 J based IL-1R2 knockout (Il1r2^−/−^) mice were used in this experiment. C57BL/6 J mice were supplied by Changzhou Cavens Laboratory Animal Co., LTD. Il1r2^−/−^mice were supplied by GemPharmatech Co., Ltd. These two kinds of mice were bred in the specific pathogen-free (SPF) animal room of Changzhou Kavins Laboratory Animal Co., LTD. The use of mice in the experiment was approved by the Ethics Committee of the Third Affiliated Hospital of Soochow University.

### Azoxymethane/dextran sodium sulfate treatment

Before the formal experiment, all the mice were first adapted to the environment for one week, and then the gender and age-matched C56BL/6 mice and Il1r2^−/−^ mice were fed together for two weeks to ensure the homogeneity of the intestinal microbiota through fecal feeding behavior, providing a more reliable and consistent basis for subsequent experiments. Then as mentioned in the literature^52^, every mice were intraperitoneally injected with 10 mg/kg AOM (Sigma Aldrich, Cat# A5486), after one week, 2.5% DSS (MP Biomedicals, Cat# 160,110) solution was given for 7 days, followed by 14 days of normal drinking water, with DSS solution and drinking water continuing for 3 cycles. Two weeks after the end of the experiment, anti-PD-1 (50 μg, G4C2 mIgG1) and anti-CTLA-4 (200 μg, 9D9) monoclonal antibody combination therapy was administered by intraperitoneal injection every 4 days for 4 times. Randomization was used during grouping to minimize potential allocation bias. Unless otherwise specified, each treatment group included at least five mice. All mice that completed the experimental protocol were included in the final analysis. Animals were excluded only when they exhibited health abnormalities unrelated to the experimental treatment, such as excessive weight loss (> 20%) or unexpected death. To minimize observer bias, investigators were blinded to the groupings during outcome assessment.

### Tumor count

The colon was obtained from the anus to the ileocecal part of the mice. The colon was cut along the longitudinal axis, and the residual feces were washed with cold PBS. The length and width of each colon polyp were measured using a ruler, and the number of tumors was recorded and counted.

### Histopathology

Each colonic polyp was carefully isolated using a surgical blade, fixed in a 10% formalin solution and embedded in a paraffin wax block. Slide thickness was set at 5 μm, and after hematoxylin and eosin (H&E) staining, detailed histological analysis was performed on paraffin-embedded sections. To ensure the objectivity and accuracy of the scoring, colonic polyps were scored histologically in a blinded manner by two experienced pathologists. The scoring criteria clearly define four major grades: normal (0), low-grade dysplasia (1), high-grade dysplasia (2), and intramucosal carcinoma (3).

### Single-cell transcriptome sequencing

At 24 h after the end of treatment, three mice per group were selected, and each colonic tumor was cut using a surgical blade close to the tumor edge, and the entire tumor was analyzed by single-cell sequencing. The viable cell ratio of the obtained single cell suspension was more than 80% after quality control, and the cell concentration was 1200–1300 cells/μL, and then the machine could be operated. In accordance with the reagent, specifications, use Chromium Next GEM Single Cell 3ʹ v3.1 (PN-1,000,121) to build scRNA-seq library. Through Chromium Controller (10 × Genomics, GCG-SR-1) for single cell capture and cDNA synthesis. After PCR amplification and purification, Qubit 4.0% & dsDNA HS Assay(Thermo, Q32854) and Agilent 4150 & High Sensitivity D5000 Tape station were used for quality control of cDNA concentration and integrity. The cDNA after quality control was subjected to the steps of fragmentation, linker ligation and Index amplification for library construction. Libraries were finally sequenced on a NovaSeq 6000(Illumina) platform to a depth of at least 20,000 reads per cell.

### Single-cell sequencing data processing

The raw files were analyzed using Cellranger (Version: 7.0.0) software from 10 × Genomics to generate a cell-specific gene expression matrix by alignment with the mouse reference genome mm10, followed by filtering correction. Subsequent downstream analysis was performed using the Seurat R package. Cells with less than 200 or more than 100,000 Unique molecular identifiers (UMIs), and cells with more than 10% mitochondrial genes and more than 30% ribosomal genes were filtered out. Harmony was used for batch correction. The NormalizeData function was used for data normalization, and FindVariableFeatures was used to calculate the mean and variance of each gene and select the top 2000 hypervariable genes for downstream analysis. The ScaleData function normalized the data for the Principal component analysis (PCA). The first 30 Principal components (PCs) were used to identify the cell subsets and the resolution was set to 0.1. The dimensionality reduction data were visualized using UMAP(Uniform manifold approximation and projection), resulting in 20,675 cells. We identified 10 major cell types based on the expression levels of marker genes characteristic of cell subsets, these cells include B cells (Cd19, Cd79a), endothelial cells (Pecam1, Vwf), epithelial cells (Epcam), fibroblasts (Col1a2, Dcn), mast cells (Cpa3), myeloid cells (Cd68, Lyz2), neutrophils (Csf3r, S100a9), plasma cells (Igha), red blood cells (Hba-a1) and T cells (Cd3e).

### Differentially expressed genes (DEG) analysis

Statistical analysis was performed using the edgeR R package to estimate the difference in expression and statistical significance of each gene between different cell populations. Significantly differentially expressed genes were screened by setting the threshold of adjusted p value (adj. *P*. value < 0.05) and log-transformed fold change (|logFoldChange|> 0.25).

### Gene set enrichment analysis

Read the differentially expressed gene data and load the necessary R packages (org.Mm.eg.db and clusterProfiler). The Gene Ontology (GO) enrichment analysis was performed using the gseGO function, and biological processes (BP) were selected as the analysis class. The KEGG and REACTOME gene sets were obtained by msigdbr package, and the differentially expressed genes were enriched by GSEA function. A bar chart is used to show the normalized enrichment score (NES) of the gene set and its significance.

### Identification of tumor cells

The human colon cancer Single-cell RNA sequencing (scRNA-seq) data GSE201349 mentioned in literature^53^ was downloaded from the Gene expression omnibus (GEO) database. Follow the single-cell analysis process described earlier, the success of normal colon and adenocarcinoma tissues were extracted epithelial cells. Differential gene expression patterns between normal and cancerous epithelial cells were identified using the FindMarkers function of the Seurat package. Then, the biomaRt R package was used to convert these differential human gene names into corresponding mouse gene names for cross-species comparison. Furthermore, VariableFeatures function was applied to set the transformed mouse genes as variable feature vectors for subsequent dimension reduction analysis, and the cells were classified into two categories: normal epithelial cells and tumor epithelial cells by clustering method.

In order to verify the accuracy of the clustering results, using infercnv R package for further analysis. This includes the preparation scRNA-seq data, create InferCNV object, and the identification of normal epithelial cells above notes for reference. We consider the CNV in parameter detection sensitivity and filtering criteria, analysis is performed after infercnv generates heat maps included a variety of graphics and data output. These results helped us to identify cells exhibiting significant CNV and to gain insight into the specific features of these variants.

### Single-cell regulatory network inference and clustering (SCENIC) analysis

GRNBoost2 was used to construct the regulatory network between transcription factors and genes, and RcisTarget was used to verify the regulatory relationship between transcription factors and target genes. The AUCell algorithm was used to evaluate the regulatory activity of each transcription factor in individual cells. The specificity of each predicted regulon for each cell type was viewed by calculating the regulon specificity score. In addition, we use scFunctions (https://github.com/FloWuenne/scFunctions/) package to calculate all regulon connection specificity index (CSI), to further evaluate the specificity of the control network.

### Statistical analysis

Experimental data using GraphPad Prism (Version: 8.0), and R–R-4.2.2, statistical analysis and drawing comparison between the two groups using Student's *t* test, weight curve comparison of the two-way ANOVA test followed by Sidak’s multiple comparisons test to adjust the risk of false positives. All data are presented as mean ± SEM, and differences were considered statistically significant when *P* < 0.05.

## Supplementary Information

Below is the link to the electronic supplementary material.

Supplementary file1 (DOC 7926 kb)

## Data Availability

Any additional information required to reanalyze the data reported in this paper is available from the lead contact upon request.
