# Supplementary material for: IL-1R2 promotes tumorigenesis and modulates the tumor immune microenvironment in colorectal cancer
Source: Cancer Immunol Immunother. 2025 Aug 6;74(9):284. doi: 10.1007/s00262-025-04138-5 (PMC12328880; doi:10.1007/s00262-025-04138-5)
Supplement: Supplementary file 1 — Supplementary file1 (DOC 7926 kb) [file 262_2025_4138_MOESM1_ESM.doc]

**
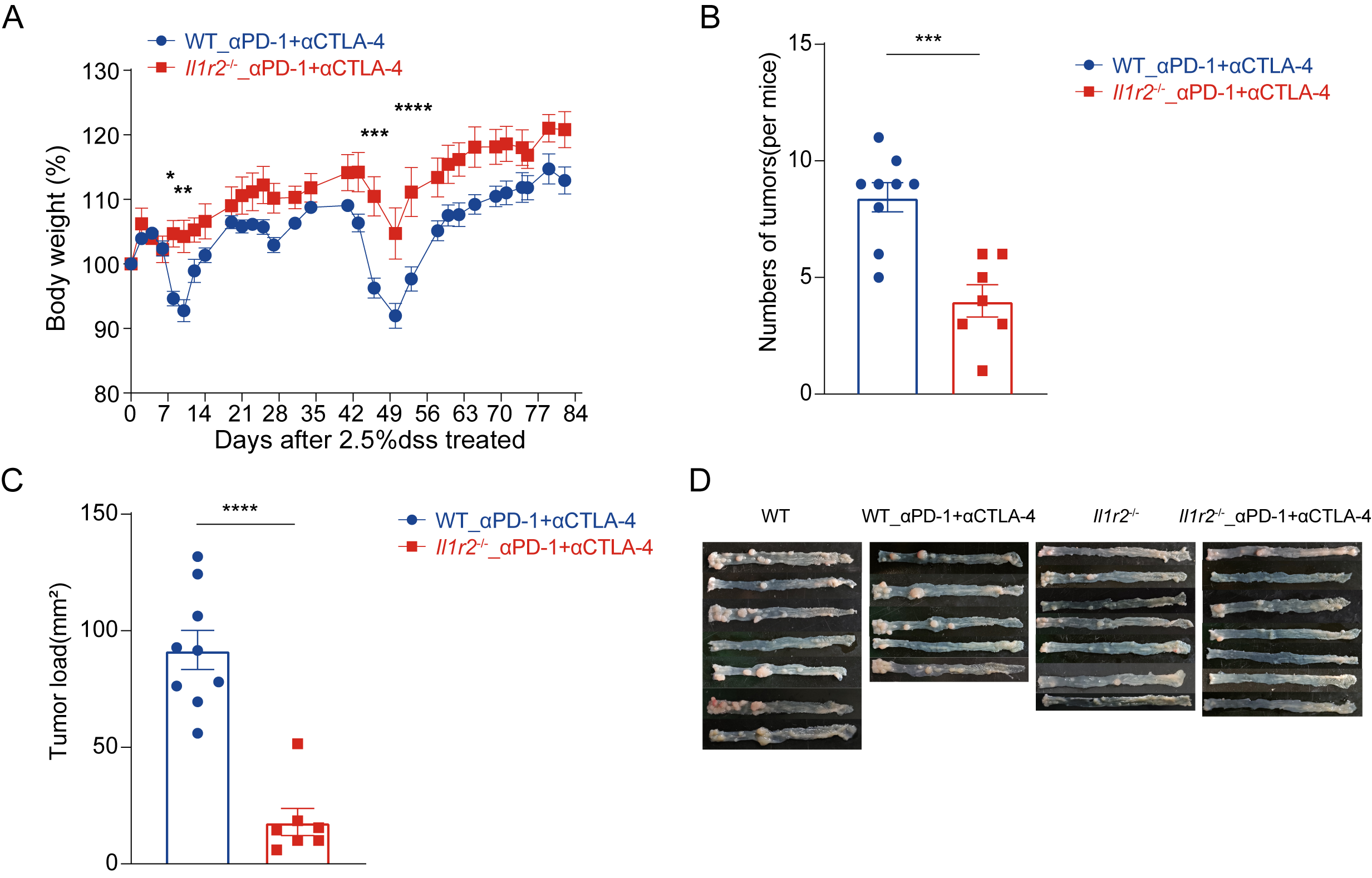
**

**Fig S1. Enhanced efficacy of immune checkpoint blockade with IL-1R2 deletion**

**A.** Body weight curves of control and Il1r2^-/-^ male mice during AOM/DSS treatment. **B.** Statistical plot of tumor number in male mice at sacrifice. **C.** Statistical plot of tumor load in male mice at sacrifice. Data are presented as Mean±SEM, *P<0.05, **P<0.01, ***P<0.001, ****P<0.0001. (WT, n = 9; Il1r2^-/-^ , n = 7). **D.** Macroscopic image of tumor in female mice at sacrifice.


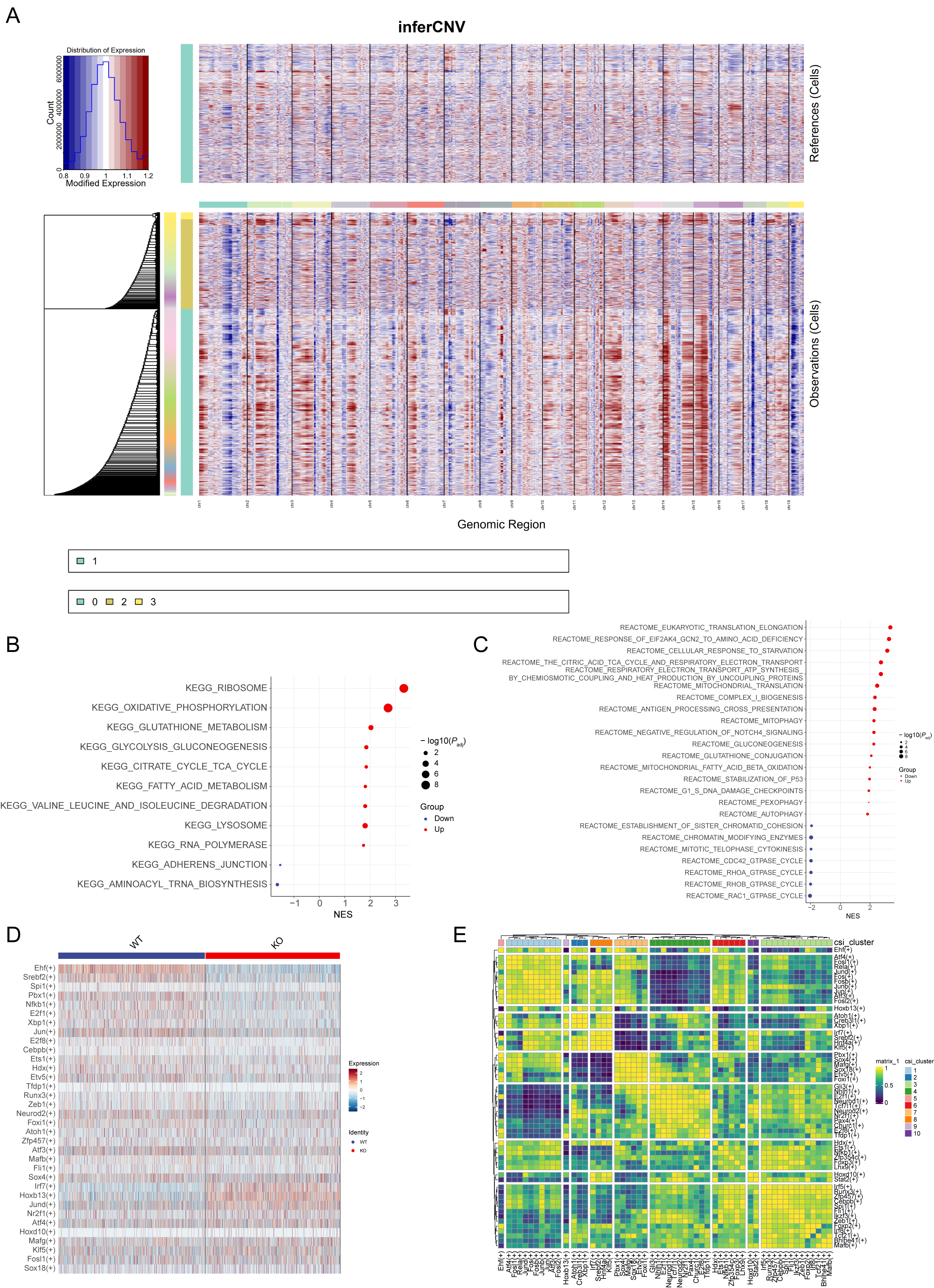


**Fig S2. Comprehensive multi-modal analysis of tumor cells**

**A.** This figure presents the inferCNV analysis used to distinguish between normal epithelial cells and tumor cells based on their copy number variations (CNVs). The heatmap shows the relative CNV intensities across different chromosomal regions, with the x-axis representing genomic coordinates and the y-axis representing individual cells. The color scale indicates the magnitude of CNVs, with red representing gains, blue representing losses, and white indicating neutral regions. **B.** Enrichment plot of KEGG pathway analysis for differentially expressed genes in tumor cells. Red represents upregulation in the KO group and blue represents upregulation in the WT group. **C.** Enrichment plot of REACTOME analysis for differentially expressed genes in tumor cells. Red represents upregulation in the KO group and blue represents upregulation in the WT group. **D.** Heat map shows a representative regulon in tumor cells. **E.** Identification of regulon modules based on the connection specificity index (CSI) matrix.


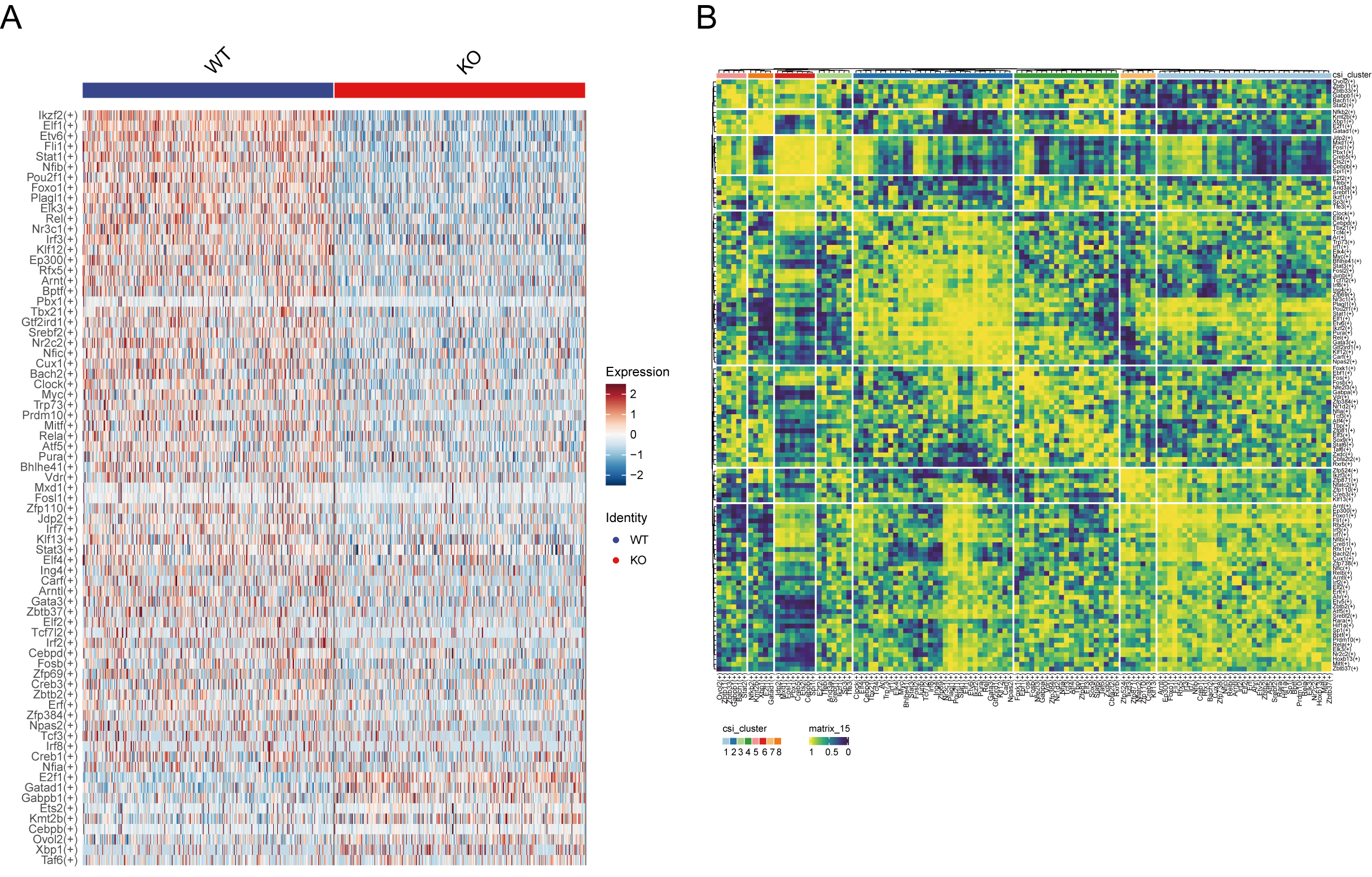


**Fig S3. Transcription factor heterogeneity SCENIC analysis of Treg cell**

**A.** Heat map shows a representative regulon in Treg cell. **B.** Identification of regulon modules based on the CSI matrix.


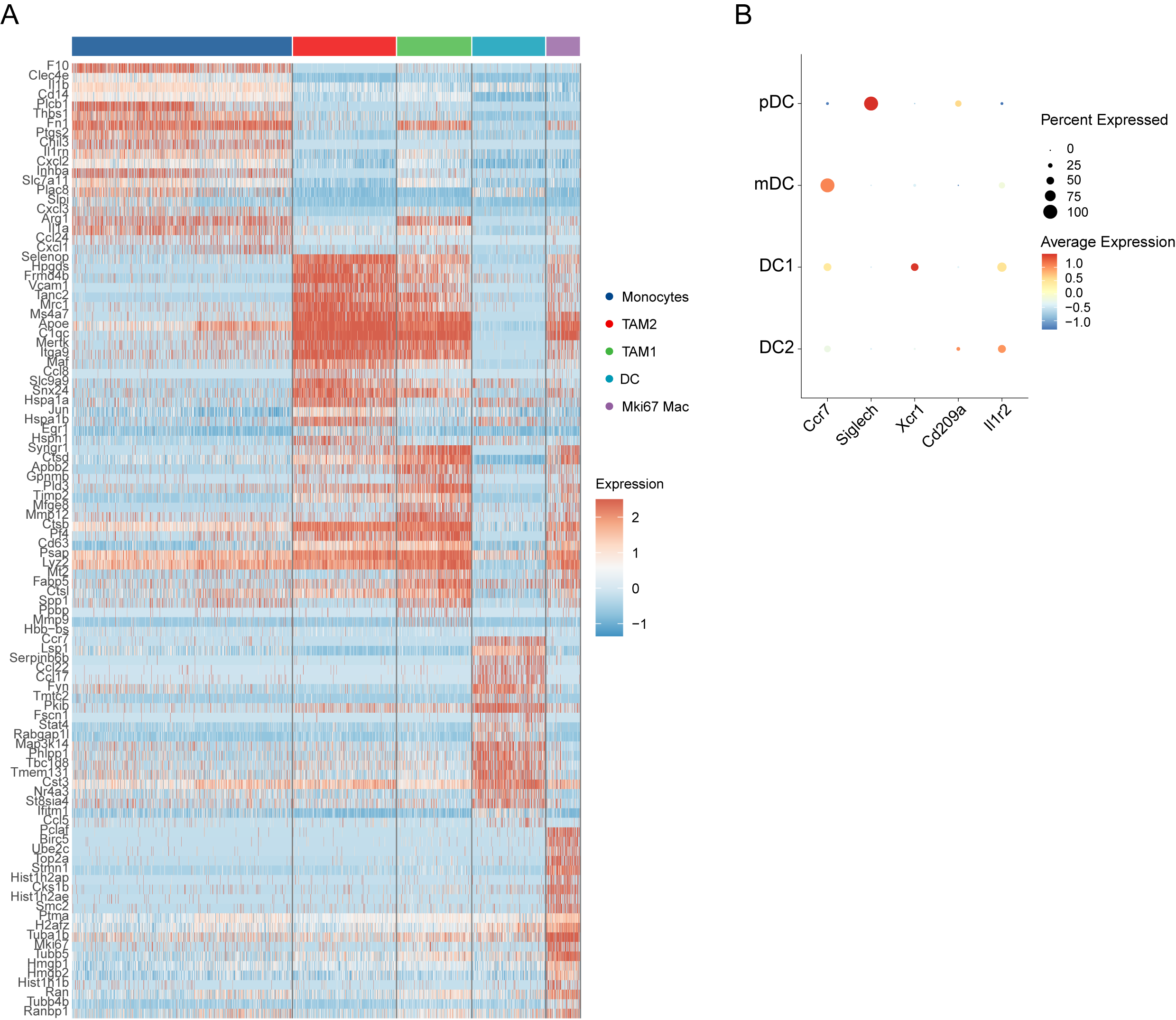


**Fig S4. Characterization of myeloid cell subsets and dendritic cell clusters**

**A.** Heatmap of characteristic genes across different myeloid cell subsets. The rows represent individual genes, and the columns represent distinct myeloid cell subsets. The color intensity reflects the level of gene expression, with higher expression shown in red and lower expression in blue. **B.** Dotplot of the expression of characteristic marker genes in dendritic cell clusters.


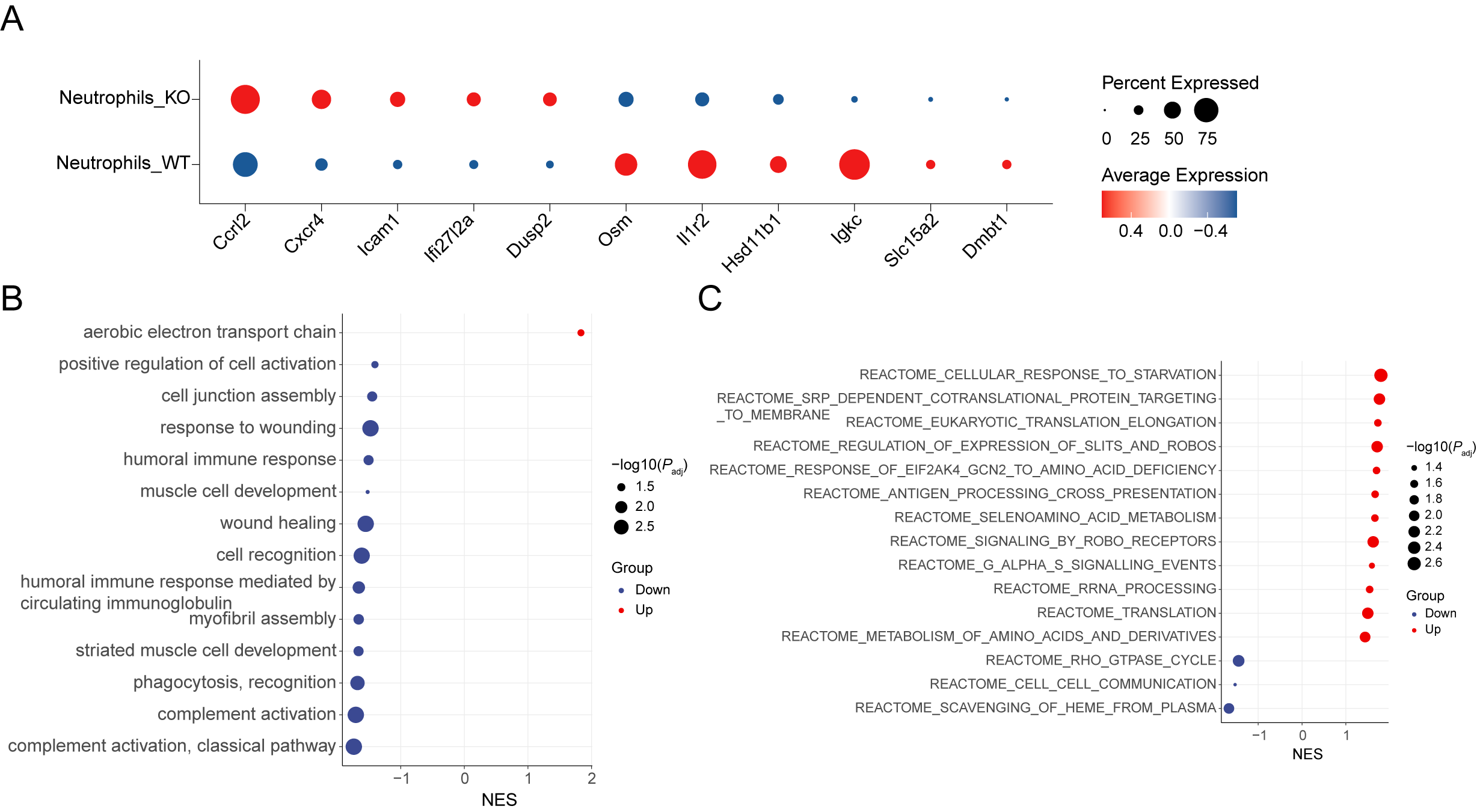


**Fig S5. Functional alterations in neutrophils**

**A.** Dotplot shows differentially expressed genes in neutrophils. **B.** Enrichment plot of GO terms for differentially expressed genes in neutrophils. Red represents upregulation in the KO group and blue represents upregulation in the WT group. **C.** Enrichment plot of REACTOME analysis for differentially expressed genes in neutrophils. Red represents upregulation in the KO group and blue represents upregulation in the WT group.
